# Characterization of Vascular Adhesion Molecules that may Facilitate Progenitor Homing in the Post-natal Mouse Thymus

**DOI:** 10.1080/10446670310001598492

**Published:** 2003-03

**Authors:** Ana Paula Lepique, Sharina Palencia, Heikki Irjala, Howard T. Petrie

**Affiliations:** 1 Memorial Sloan-Kettering Cancer Center Box 341, 1275 York Avenue New York NY 10021 USA; 2 Medicity Research Laboratory University of Turku Turku Finland; 3 Weill Graduate School of Medical Sciences of Cornell University New York NY 10021 USA

## Abstract

T cell progenitors derive from the bone marrow but must migrate via bloodstream to the thymus in order to differentiate. The mechanism by which the thymus recruits progenitors from the blood is unknown. It is known, however, that there are receptive and refractory periods for progenitor recruitment and that when cells are imported, they enter the thymus through post-capillary venules. Therefore, recruitment is an active process temporally and spatially regulated. In order to characterize the mechanism of recruitment, we evaluated vascular signals known to regulate leukocyte extravasation, with respect to their intrathymic location and temporal fluctuations. We find that CD34, MECA79, VCAM-1, ICAM-1 and VAP-1 are all expressed in thymic blood vessels. MECA79 and VAP-1 appear to be specific for post-capillary venules, while ICAM-1 and VCAM-1 are also found on intrathymic stromal cells. MAdCAM is also expressed in the thymus, but is not associated with vascular tissues. Only MECA79 is upregulated during recruitment peaks, suggesting a role for this molecule in the periodicity of recruitment. Together, these studies reveal potential roles for l-selectin ligands, VCAM-1, ICAM-1 and VAP-1 in progenitor recruitment to the thymus, and implicate the presence of other periodic signals, such as chemokines and cytokines, that cooperate to execute this essential function.


*Clinical & Developmental Immunology*, March 2003, Vol. 10 (1), pp. 27–33

Taylor & Francis healthsciences

### Characterization of Vascular Adhesion Molecules that may Facilitate Progenitor Homing in the Post-natal Mouse Thymus

ANA PAULA LEPIQUE[a], SHARINA PALENCIA[a], HEIKKI IRJALA[b] and HOWARD T. PETRIE[a,c,*]

[a]*Memorial Sloan-Kettering Cancer Center, Box 341, 1275 York Avenue, New York, NY 10021, USA;* [b]*Medicity Research Laboratory, University of Turku, Turku, Finland;* [c]*Weill Graduate School of Medical Sciences of Cornell University, New York, NY 10021, USA*

T cell progenitors derive from the bone marrow but must migrate via bloodstream to the thymus in order to differentiate. The mechanism by which the thymus recruits progenitors from the blood is unknown. It is known, however, that there are receptive and refractory periods for progenitor recruitment and that when cells are imported, they enter the thymus through post-capillary venules. Therefore, recruitment is an active process temporally and spatially regulated. In order to characterize the mechanism of recruitment, we evaluated vascular signals known to regulate leukocyte extravasation, with respect to their intrathymic location and temporal fluctuations. We find that CD34, MECA79, VCAM-1, ICAM-1 and VAP-1 are all expressed in thymic blood vessels. MECA79 and VAP-1 appear to be specific for post-capillary venules, while ICAM-1 and VCAM-1 are also found on intrathymic stromal cells. MAdCAM is also expressed in the thymus, but is not associated with vascular tissues. Only MECA79 is upregulated during recruitment peaks, suggesting a role for this molecule in the periodicity of recruitment. Together, these studies reveal potential roles for L-selectin ligands, VCAM-1, ICAM-1 and VAP-1 in progenitor recruitment to the thymus, and implicate the presence of other periodic signals, such as chemokines and cytokines, that cooperate to execute this essential function.

*Keywords*: Adhesion; Extravasation; Progenitor homing; Thymus

### INTRODUCTION

T cell progenitors are generated in the bone marrow and travel through the blood stream to enter the thymus, where they develop into mature T cells. However, there is little published information regarding the molecular mechanisms by which progenitors are recruited to the post-natal thymus. Our laboratory and others have shown that T cell progenitors enter the thymus through large blood vessels near the cortico-medullary junction (Brumby and Metcalf, 1967; Brahim and Osmond, 1970; Kyewski, 1987; Dunon *et al.*, 1997; Lind *et al.*, 2001). This recruitment of T cell progenitors to the thymus is an active phenomenon (Foss *et al.*, 2001; 2002); the thymus is receptive to progenitor entry for approximately 1 week, followed by a 3-week refractory period. The availability of undefined intrathymic stromal niches is thought to regulate the status of cell entry, such that once intrathymic sites are saturated with prothymocytes, new progenitors are not able to enter until the intrathymic progenitors move on and leave the niches empty (Foss *et al.*, 2001; 2002). Together, the data on the restricted location of cell entry, and the periodicity of this process, indicate that progenitor recruitment to the thymus is regulated both spatially and temporally.

The process of leukocyte extravasation is fairly well characterized in secondary lymphoid organs and sites of chronic inflammation (Campbell and Butcher, 2000; Worthylake and Burridge, 2001). The first steps in the process are rolling and tethering, mediated by selectins and their ligands. L-Selectin ligands, which include CD34 (Baumheter *et al.*, 1993), GlyCAM (Mebius *et al.*, 1993) and MadCAM (Berg *et al.*, 1993) can be modified post-translationally with carbohydrates such as MECA79 (Hemmerich *et al.*, 1994), which are necessary for optimal ligand binding. Subsequently, integrins are required to mediate firm adhesion, and expression of their ligands, including the canonical ligands MAdCAM-1 (de Chateau *et al.*, 2001; Salas *et al.*, 2002), ICAM-1 (Springer, 1994; Salas *et al.*, 2002) and VCAM-1 (Weber and Springer, 1998), are required for this process. Some of these, including ICAM-1 and VCAM-1 are upregulated in response to pro-inflammatory cytokines and chemokines (Osborn *et al.*, 1989; Xu *et al.*, 1994). In addition, Vascular Adhesion Protein-1 (VAP-1) is a member of a newly described class of adhesion molecules that differ in the expression of both an adhesion domain and a catalytic domain. VAP-1 has a known role in leukocyte extravasation (for a review see, Salmi and Jalkanen, 2001),

*Corresponding author. Address: Memorial Sloan-Kettering Cancer Center, Box 341, 1275 York Avenue, New York, NY 10021, USA. Tel.: +1-212-639-2149. Fax: +1-212-794-4019. E-mail: petrieh@mskcc.org

ISSN 1740-2522 print/ISSN 1740-2530 online © 2003 Taylor & Francis Ltd
DOI: 10.1080/10446670310001598492

and is upregulated by inflammatory cytokines such IL1 and TNFα (Arvilommi *et al.*, 1997).

In the present manuscript, we characterize expression of some of the canonical mediators of leukocyte extravasation (described above) in thymic progenitor seeding. We show that post-capillary venules in the post-natal thymus express a number of adhesion molecules related to rolling, tethering and firm adhesion, suggesting a potential role for these in the extravasation process. Of interest, only one of these, MECA79, is regulated with respect to the periodicity of progenitor recruitment, implicating the regulation of other signaling molecules, possibly including chemokines and/or inflammatory cytokines, in regulating the periodicity of this process.

### MATERIALS AND METHODS

### Mice and Antibodies

C57BL/6 male mice were purchased from NCI and kept under SPF conditions at Memorial Sloan-Kettering Cancer Center. Rat anti-mouse MAdCAM-1 was a gift from Dr Klaus Ley, University of Virgina; Rat anti-mouse CD34 was a kind from Dr Steven Nimer, MSKCC; Rat anti-mouse PNAd (MECA79) was purchased from BD Pharmingen; Hamster anti-mouse ICAM-1 (Pharmingen) was a gift from Dr Ralph Steiman, Rockefeller University; Rat anti-mouse VCAM-1 (clone M/K-2.7) was generated in the investigator's laboratory; Rat anti-mouse VAP-1 (Monoclonal antibody 7–188 against mouse VAP-1 molecule was produced in rat, detailed description will be published separately); biotin conjugated rabbit anti-rat IgG was purchased from Vector Laboratories; biotin conjugated rabbit anti-rat IgG + IgM and biotin conjugated goat anti-Armenian hamster IgG were purchased from Jackson ImmunoResearch.

### Tissue Sectioning and Freezing

Thymuses were harvested from mice and lobes separated with thin forceps. Corresponding lobes from mice of 4, 5, 7 and 9 weeks of age were immediately frozen side by side in the same mold in OCT Tissue Tech reagent (VWR). Transverse frozen sections of 5 μm thickness were taken at 2, 3, 4, 5 and 6 mm depths relative to the anterior–posterior axis (the thymus is generally 7 mm from anterior to posterior). Sections were stored at −20°C in a desiccated box until use.

### Immunohistochemistry

Dessicated frozen sections were fixed in ice-cold acetone for 30 min. Endogenous peroxidase activity was quenched by incubating slides for 30 min in a solution of 1 mM $NaN_3$, 10 mM glucose, and 1 U/ml of glucose oxidase (Calbiochem). Incubation in Avidin/biotin Blocking Kit (Vector laboratories) for endogenous biotin quenching, followed by 5% Fetal Bovine Serum (FBS) incubation for blocking non-specific binding were performed before antibody incubations. All antibody incubations were performed for 30 min at room temperature. Single-color histochemical detection was performed using an avidin-peroxidase conjugate system (VectaStain Elite; Vector Laboratories), and antibody-enzyme complex was visualized with 3′3′-diaminobenzidene (DAB; Sigma-Aldrich) and 0.1% $H_2O_2$. Incubation times were carefully monitored to prevent saturation, thus favoring visualization of differences in expression levels. Sections were counterstained using Mayer's hematoxylin (Fisher Scientific), dehydrated through graded ethanol, cleared in Hemo-De (Fisher Scientific), and cover slipped using Permount (Fisher Scientific) for examination. Photomicrographs were taken using an Olympus BX40 microscope and Pixera Studio version 1.2 software (Pixera Corporation).

### RESULTS

### MECA79 is Expressed in Post-capillary Venules in the Adult Mouse Thymus

When expression of MECA79, one of the carbohydrate structures that modify ligands for L-selectin, was examined by immunohistochemistry, it was found to be expressed mainly on post-capillary venules deep in the cortex or in the cortico-medullary junction (Fig. 1). Expression of MECA79 appeared to be higher in tissues from animals at 5 and 9 weeks of age (peak of progenitor recruitment), and weaker in tissue from animals at 4 and 7 weeks of age (refractory periods). Proteins modified by MECA79, such as CD34 (Suzuki *et al.*, 1996), are not restricted to post-capillary venules at the cortico-medullary junction, but rather are expressed by all vascular elements in the mouse thymus. Together, these findings indicate that the signals that regulate both the periodicity and the location of progenitor recruitment to the thymus may involve post-translational changes in scaffold proteins, rather than alterations in adhesion molecule synthesis *per se*.

### VCAM-1 and ICAM-1 are Expressed by both Thymic Stromal Cells and Post-capillary Venules

Figures 2 and 3 show immunohistochemical analysis of VCAM-1 and ICAM-1, respectively. VCAM-1 was found to be expressed in venules near the cortico-medullary junction, as well as in the deep cortex and medulla, and was also present on some stromal cells (Fig. 2). However, the levels of VCAM-1 on vascular cells appeared to be significantly higher than those found on stromal cells. No differences in VCAM-1 expression were found in thymic sections from animals at different ages (4–9 weeks), indicating that while VCAM-1 may play a role in facilitating progenitor homing to the thymus, it does not appear to be involved in the periodicity of this event.

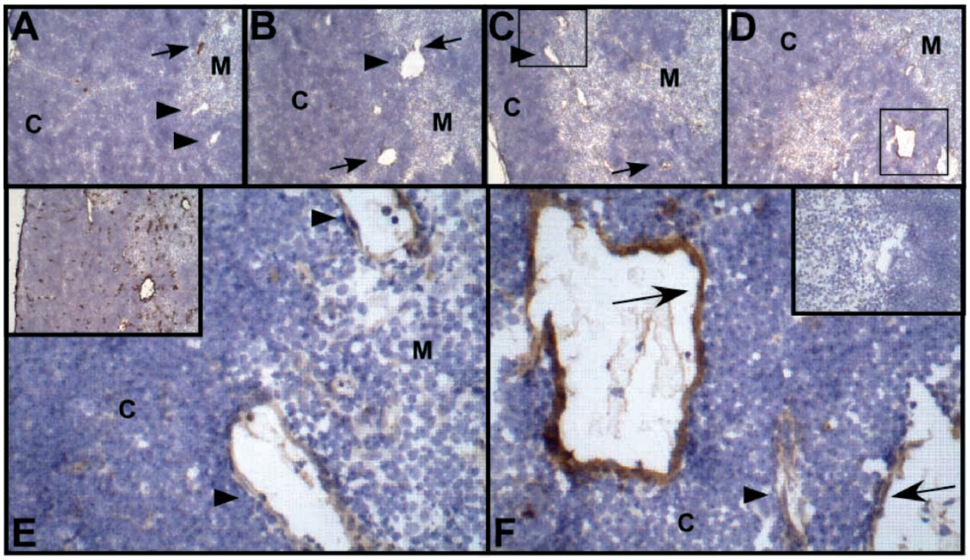

FIGURE 1 MECA79 is expressed on specific vascular structures in the mouse thymus. MECA79 was examined by immunohistochemistry on thymic sections of animals at 4 weeks (A), 5 weeks (B), 7 weeks (C and E) and 9 weeks (D and F) of age; these intervals encompass one complete cycle of progenitor recruitment (see text for further details). MECA79 expression was found on most venules near the sites of progenitor entry, and was most abundant at peak periods of progenitor recruitment (i.e. 5 and 9 weeks), and lower during refractory periods (4 and 7 weeks). Regions outlined by rectangles in panels C and D are enlarged in panels E and F, respectively. The inset on panel E shows thymic expression of CD34, one of the sialomucins that can be modified by MECA79; the inset on panel F is an isotype-matched antibody control. Original magnifications: A through D, 100 × ; E and F, 400 × . M = medulla; C = cortex.

Similar to the distribution of VCAM-1, ICAM-1 was expressed by blood vessels deep in the tissue, both in the cortex and in the medulla (Fig. 3). Again, there appears to be differential expression of ICAM-1 among the blood vessels present. Some of the larger ones displayed a stronger, sharper staining in the luminal aspect of the vessel (Fig. 3B, arrow), while other smaller vessels presented a more homogenous and weaker staining. Some stromal cells also expressed ICAM-1, both in the cortex and in the medulla. It is difficult to say whether the apparently higher expression by stromal cells in the medulla is truly higher than it is in the cortex (see Fig. 3B, for details), or whether the apparent difference is a consequence of the differences in cell density in each compartment. In any case, ICAM-1 is present, and its expression does not appear to vary in relation to animal age, suggesting that like VCAM-1, it may play a role in progenitor entry into the thymus, although not in the periodicity of this process.

### VAP-1 Expression is Restricted to Specific Venules found near the Sites of Progenitor Homing

Analysis of VAP-1 expression showed that it was restricted to blood vessels, and more precisely, to a relatively few venules found near the corticomedullary junction (Fig. 4). VAP-1 expression was not homogenous in most cases, showing a general bias towards the cortical aspects of the vessels where it was expressed. Venules within the medullary compartment did not express VAP-1,

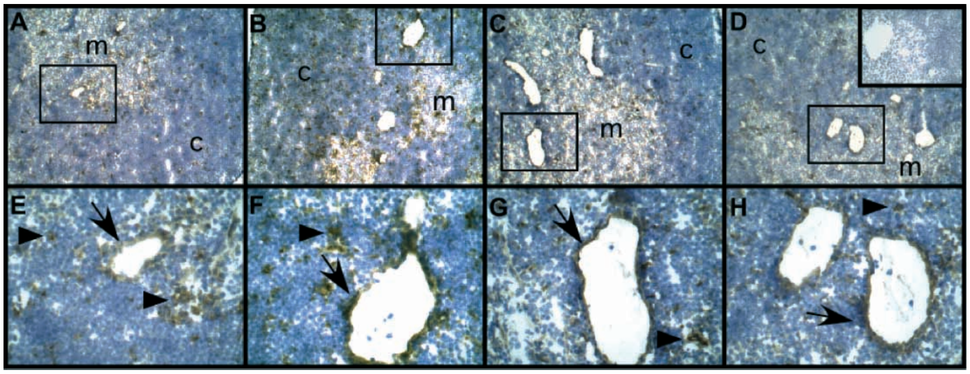

FIGURE 2 VCAM expression on thymic stromal cells and endothelial cells of postcapillary venules. VCAM expression was examined by immunohistochemistry on thymuses from animals at 4 weeks (A and D), 5 weeks (B and E), 7 weeks (C and F) and 9 weeks (D and H) of age. Regions defined on the low power images (A–D, 100 × original magnification) are enlarged in panels E–F (400 × original magnification). Positive staining was observed at a relatively higher level on blood vessels (arrows) than on stromal cells (arrowheads). Inset in panel D shows an isotype-matched antibody control. M = medulla; C = cortex.

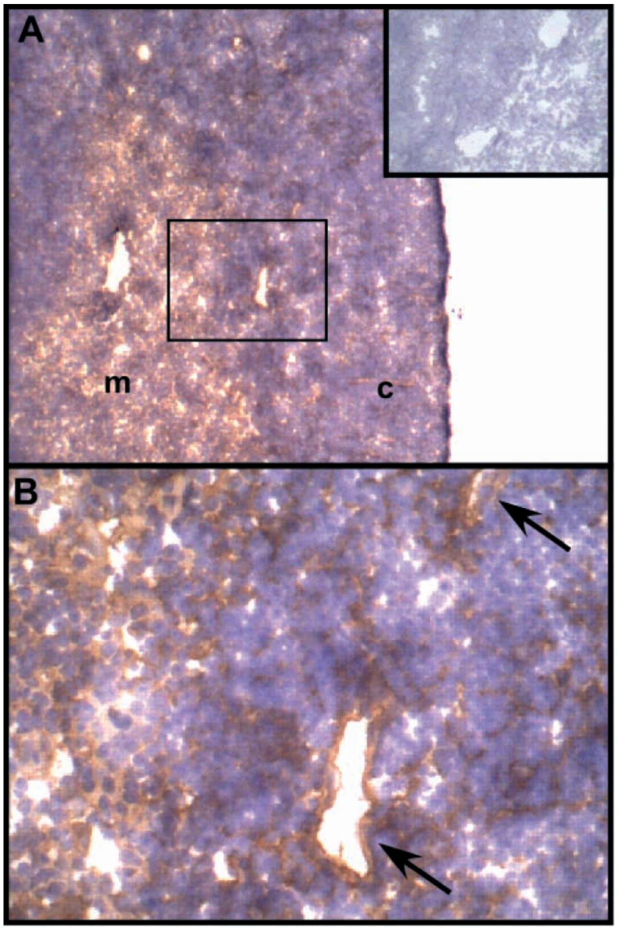

FIGURE 3 ICAM-1 is expressed by endothelial and stromal cells in the mouse thymus. Panel A shows a low magnification (100×) image of ICAM-1 immunohistochemical staining; inset shows an isotype-matched antibody control. Staining was strongest in the medulla and lower in the cortex, but was basically expressed throughout the thymus. No differences were found with respect to thymic homing potential (see text). The area indicated by a rectangle is enlarged in a high power (400× original magnification) image in B. A few post-capillary vessels are indicated by arrows; some of these show a distinction in the intensity of staining between luminal and abluminal aspects (see text). M = medulla, C = cortex.

nor did cortical capillaries. Nonetheless, VAP-1 expression did not appear to change during peak or refractory periods of progenitor homing to the thymus. These results strongly implicate VAP-1 in the process of recruiting progenitors to the thymus, but again, VAP-1 does not appear to be related to the periodicity of entry.

### MAdCAM-1 is Expressed in the Thymus, but not by Endothelial Cells

MAdCAM-1 is an adhesion molecule most frequently involved in rolling and tethering of leukocytes in the circulation. In the thymus, MAdCAM-1 is expressed, but it appears to be restricted to a relatively few cells found within the medulla proper (Fig. 5). Based on their morphology, these appear to be stromal cells, although they are clearly not present in sufficient number to include all medullary stroma. Blood vessels in the thymus are

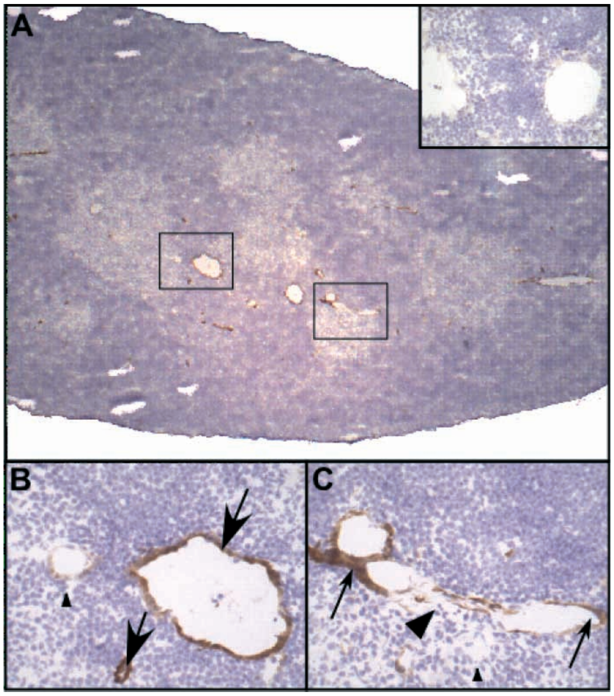

FIGURE 4 VAP-1 expression is restricted to a subset of venules found near the sites of progenitor homing. Panel A shows a low power image (40× original magnification) with an anti-VAP-1 antibody. Inset is an isotype-matched antibody control; rectangles show regions of interest enlarged in Panels B and C (400× original magnification). Parts of some venules near the cortico-medullary junction express high levels of VAP-1 (arrows), while others are very weakly stained or completely absent (arrowheads). M = medulla, C = cortex.

categorically negative for MAdCAM-1, even though the same concentrations of antibody readily detected MAdCAM-1 in sections of mesenteric lymph node. Thus, although MAdCAM-1 is expressed in the thymus, it does not appear to be associated with blood vessels and is therefore unlikely to be involved in any aspect of progenitor recruitment.

### DISCUSSION

In the present manuscript, we show that blood vessels in the post-natal thymus express a variety of adhesion molecules that facilitate leukocyte extravasation in secondary lymphoid organs or in inflamed tissue. Only one of these, MECA79, fluctuated temporally with respect to periods of progenitor recruitment, being highest during homing peak periods. MECA79 has previously been shown to be present in the medulla of the AKR hyperplastic thymus (Michie *et al.*, 1995). Here, we find it to be expressed by postcapillary venules at the sites where progenitors are known to enter the post-natal thymus. Since CD34, which is one of the sialomucins that can be modified by MECA79, is expressed on all thymic vasculature, this finding suggests that post-translational modification, rather than protein synthesis, may be a critical factor regulating the periodicity of progenitor

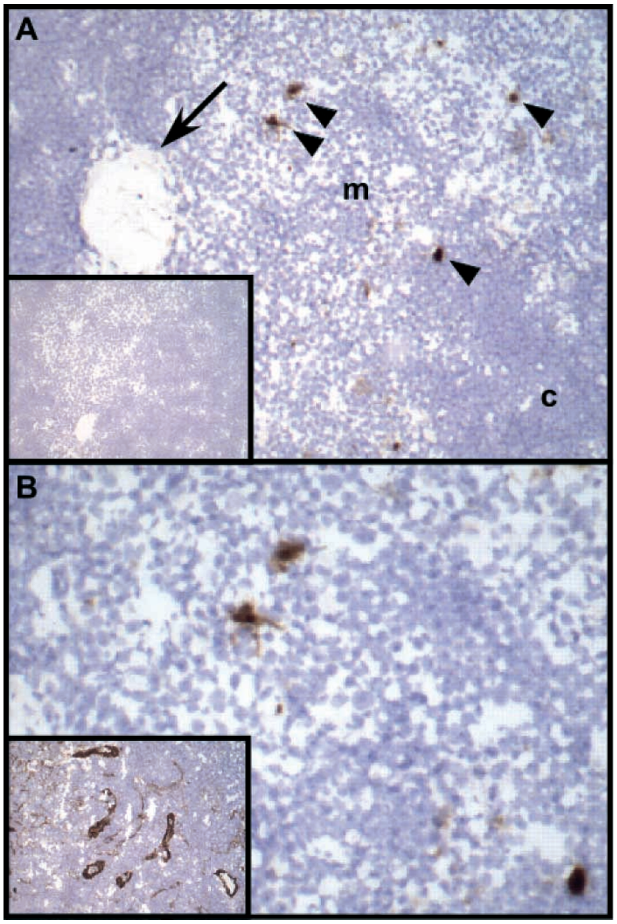

FIGURE 5 MAdCAM-1 is expressed in the thymus, but not by endothelial cells. MAdCAM-1 expression was examined by immunohistochemistry on thymus. Cells expressing MAdCAM-1 (arrowheads) are found within medullary (M) areas, but not in the cortex (C). For comparison, the location of a large post-capillary venule is indicated by an arrow. The inset shows an isotype-matched antibody control. Panel B shows a high power image of the central region in Panel A, illustrating the morphology of MAdCAM-1-expressing cells. The inset on Panel B shows MAdCAM-1 staining on mesenteric lymph node. Thymuses from animals of all ages tested showed similar results. Original magnifications: Panel A, 200 ×, Panel B, 400 ×.

recruitment to the thymus. This, in turn, suggests that the feedback induced by niche occupancy in the thymus (Foss *et al.*, 2001; 2002) may activate stromal and/or endothelial cells to upregulate enzymes such as 6-O GlcNAc sulfotransferase, which are required for such post-translational modifications (Hemmerich *et al.*, 2001). In any case, our results suggests that L-selectin ligands may play a role in progenitor recruitment to the thymus, and that post-translational modification of L-selecting ligands may be involved in regulating the periodicity of this event. MAdCAM-1, another sialomucin that can be modified by MECA79, was not found in association with blood vessels in the adult thymus, but others have reported it to be expressed by thymic venules at around the time of birth (Iizuka *et al.*, 2000), suggesting that it might play a role in progenitor recruitment during embryonic development or in the first perinatal wave of T cell production (Dunon *et al.*, 1993).

Although they did not fluctuate with respect to periods of homing activity, other adhesion markers were found to be expressed by thymic vascular cells as well. Both ICAM-1 and VCAM-1 were found in thymic blood vessels, including post-capillary venules at the cortico-medullary junction, indicating a potential role for these molecules in leukocyte extravasation into the thymus. We also found both of these to be expressed by thymic stromal cells. We and others have previously found VCAM-1 expression by thymic stroma (Salomon *et al.*, 1997; Prockop *et al.*, 2002), and we have shown it to be a critical ligand for transcortical migration of intrathymic progenitors (Prockop *et al.*, 2002). Likewise, ICAM-1 has been shown by others to be expressed on various thymocytes, including thymocytes themselves (Reiss and Engelhardt, 1999), nurse cells (Cordes *et al.*, 1997; Oliveira-dos-Santos *et al.*, 1997), and other stromal cells (Reiss and Engelhardt, 1999; Lucas and Germain, 2000). While we did not observe any differences in ICAM-1 expression on vascular cells with respect to the periodicity of progenitor homing, we did observe a difference in the pattern of staining on different types of vessels (see "Results" section), although the implications of this are presently unknown.

Although they did not fluctuate with respect to homing activity, the expression patterns shown by VAP-1 in the thymus are particularly interesting for other resons. VAP-1 was restricted to only a very few venules, and all of these were near the sites of progenitor entry at the cortico-medullary boundary. Furthermore, in venules that actually span the border of cortical and medullary regions, the distribution of VAP-1 was asymmetric with higher expression towards the cortical aspect of the vessel, whereas in deep cortical venules that do not contact the medulla, distribution was homogenous around the perimeter. Thus, VAP-1, which could be involved in rolling/tethering, diapedesis, or both (Salmi and Jalkanen, 2001), displays a pattern of expression that is consistent with directing cell entry specifically in the region of the peri-medullary cortex, as previously shown (Lind *et al.*, 2001). Further, the fact that VAP-1 is only expressed on a small subset of venules in this region is consistent with the small number of cells that enter the thymus at any one time (Shortman *et al.*, 1990). Thus, VAP-1 appears to have significant potential as a candidate regulating the specificity of progenitor homing to the post-natal thymus via the blood.

Overall, it is interesting to note that with the exception of MECA79, none of the other adhesion molecules tested here were modulated with respect to the status of new progenitor recruitment by the thymus. It is possible that fluctuations in MECA79 expression are sufficient to regulate the periodicity of this process. However, in other tissues where leukocyte recirculation does not occur constitutively, other factors, such as inflammatory cytokines, are generally required to facilitate the extravasation process (Smith *et al.*, 1991; Vaday *et al.*, 2001). Chemokines are also heavily implicated, both in inflammed tissue and in tissues where leukocyte recirculation is constitutive (Bargatze and Butcher, 1993; Rossi and Zlotnik, 2000; Mackay, 2001). Since adhesion molecules expressed by thymic endothelial cells are also found on blood vessels in a variety of other tissues, it is likely that additional signals are required to impart specificity to the thymic homing process. Thus, although critical reagents are generally lacking at present, characterization of cytokine and chemokine expression *in situ* at the sites of progenitor entry is likely to yield important information about the recruitment process. Together with characterization of adhesion molecule expression, such as presented here, such data will provide important insights into the regulation of the critical and poorly understood process of progenitor recruitment to the thymus in the steady state.

### Acknowledgements

The authors would like to thank Dr Klaus Ley, University of Virgina, for anti-MAdCAM antibody; Dr Steven Nimer, MSKCC, for anti-CD34 antibody; Dr Ralph Steiman, Rockefeller University, for anti-ICAM-1 antibody; Dr Andrew Farr, MSKCC, for the peroxidase quenching protocol. This work was supported by PHS grants A133940 (to H.T. Petrie) and CA08748 (to MSKCC). A.P. Lepique was supported by fellowship from CNPq, 200722/01-8 PDE, Brazil.

### References

Arvilommi, A.M., Salmi, M. and Jalkanen, S. (1997) "Organ-selective regulation of vascular adhesion protein-1 expression in man", *Eur. J. Immunol.* **27**, 1794–1800.

Bargatze, R.F. and Butcher, E.C. (1993) "Rapid G protein-regulated activation event involved in lymphocyte binding to high endothelial venules", *J. Exp. Med.* **178**, 367–372.

Baumheter, S., Singer, M.S., Henzel, W., *et al.* (1993) "Binding of L-selectin to the vascular sialomucin CD34", *Science* **262**, 436–438.

Berg, E.L., McEvoy, L.M., Berlin, C., Bargatze, R.F. and Butcher, E.C. (1993) "L-Selectin-mediated lymphocyte rolling on MAdCAM-1", *Nature* **366**, 695–698.

Brahim, F. and Osmond, D.G. (1970) "Migration of bone marrow lymphocytes demonstrated by selective bone marrow labeling with thymidine-H3", *Anat. Rec.* **168**, 139–159.

Brumby, M. and Metcalf, D. (1967) "Migration of cells to the thymus demonstrated by parabiosis", *Proc. Soc. Exp. Biol. Med.* **124**, 99–103.

Campbell, J.J. and Butcher, E.C. (2000) "Chemokines in tissue-specific and microenvironment-specific lymphocyte homing", *Curr. Opin. Immunol.* **12**, 336–341.

de Chateau, M., Chen, S., Salas, A. and Springer, T.A. (2001) "Kinetic and mechanical basis of rolling through an integrin and novel Ca2+-dependent rolling and Mg2+-dependent firm adhesion modalities for the alpha 4 beta 7-MAdCAM-1 interaction", *Biochemistry* **40**, 13972–13979.

Cordes, U., Pedersen, M., Bastholm, L., Nielsen, M. and Werdelin, O. (1997) "Murine thymic nurse cells express ICAM-1 on caveolar and vacuolar membranes", *Scand. J. Immunol.* **46**, 344–348.

Dunon, D., Ruiz, P. and Imhof, B.A. (1993) "Pro-T cell homing to the thymus", *Curr. Top. Microbiol. Immunol.* **84**, 139–150.

Dunon, D., Courtois, D., Vainio, O., *et al.* (1997) "Ontogeny of the immune system: gamma/delta and alpha/beta T cells migrate from thymus to the periphery in alternating waves", *J. Exp. Med.* **186**, 977–988.

Foss, D.L., Donskoy, E. and Goldschneider, I. (2001) "The importation of hematogenous precursors by the thymus is a gated phenomenon in normal adult mice", *J. Exp. Med.* **193**, 365–374.

Foss, D.L., Donskoy, E. and Goldschneider, I. (2002) "Functional demonstration of intrathymic binding sites and microvascular gates for prothymocytes in irradiated mice", *Int. Immunol.* **14**, 331–338.

Hemmerich, S., Butcher, E.C. and Rosen, S.D. (1994) "Sulfation-dependent recognition of high endothelial venules (HEV)-ligands by L-selectin and MECA 79, and adhesion-blocking monoclonal antibody", *J. Exp. Med.* **180**, 2219–2226.

Hemmerich, S., Bistrup, A., Singer, M.S., *et al.* (2001) "Sulfation of L-selectin ligands by an HEV-restricted sulfotransferase regulates lymphocyte homing to lymph nodes", *Immunity* **15**, 237–247.

Iizuka, T., Tanaka, T., Suematsu, M., *et al.* (2000) "Stage-specific expression of mucosal addressin cell adhesion molecule-1 during embryogenesis in rats", *J. Immunol.* **164**, 2463–2471.

Kyewski, B.A. (1987) "Seeding of thymic microenvironments defined by distinct thymocytestromal cell interactions is developmentally controlled", *J. Exp. Med.* **66**, 520–538.

Lind, E.F., Prockop, S.E., Porritt, H.E. and Petrie, H.T. (2001) "Mapping precursor movement through the postnatal thymus reveals specific microenvironments supporting defined stages of early lymphoid development", *J. Exp. Med.* **194**, 127–134.

Lucas, B. and Germain, R.N. (2000) "Opening a window on thymic positive selection: developmental changes in the influence of cosignaling by integrins and CD28 on selection events induced by TCR engagement", *J. Immunol.* **165**, 1889–1895.

Mackay, C.R. (2001) "Chemokines: immunology's high impact factors", *Nat. Immunol.* **2**, 95–101.

Mebius, R.E., Dowbenko, D., Williams, A., Fennie, C., Lasky, L.A. and Watson, S.R. (1993) "Expression of GlyCAM-1, an endothelial ligand for L-selectin, is affected by afferent lymphatic flow", *J. Immunol.* **151**, 6769–6776.

Michie, S.A., Streeter, P.R., Butcher, E.C. and Rouse, R.V. (1995) "L-Selectin and alpha 4 beta 7 integrin homing receptor pathways mediate peripheral lymphocyte traffic to AKR mouse hyperplastic thymus", *Am. J. Pathol.* **147**, 412–421.

Oliveira-dos-Santos, A.J., Rieker-Geley, T., Recheis, H. and Wick, G. (1997) "Murine thymic nurse cells and rosettes: analysis of adhesion molecule expression using confocal microscopy and a simplified enrichment method", *J. Histochem. Cytochem.* **45**, 1293–1297.

Osborn, L., Hession, C., Tizard, R., *et al.* (1989) "Direct expression cloning of vascular cell adhesion molecule 1, a cytokine-induced endothelial protein that binds to lymphocytes", *Cell* **59**, 1203–1211.

Prockop, S.E., Palencia, S., Ryan, C.M., Gordon, K., Gray, D. and Petrie, H.T. (2002) "Stromal cells provide the matrix for migration of early lymphoid progenitors through the thymic cortex", *J. Immunol.* **169**, 4354–4361.

Reiss, Y. and Engelhardt, B. (1999) "T cell interaction with ICAM-1-deficient endothelium *in vitro*: transendothelial migration of different T cell populations is mediated by endothelial ICAM-1 and ICAM-2", *Int. Immunol.* **11**, 1527–1539.

Rossi, D. and Zlotnik, A. (2000) "The biology of chemokines and their receptors", *Annu. Rev. Immunol.* **18**, 217–242.

Salas, A., Shimaoka, M., Chen, S., Carman, C.V. and Springer, T. (2002) "Transition from rolling to firm adhesion is regulated by the conformation of the I domain of the integrin lymphocyte function-associated antigen-1", *J. Biol. Chem.* **277**, 50255–50262.

Salmi, M. and Jalkanen, S. (2001) "VAP-1: an adhesin and an enzyme", *Trends Immunol.* **22**, 211–216.

Salomon, D.R., Crisa, L., Mojcik, C.F., Ishii, J.K., Klier, G. and Shevach, E.M. (1997) "Vascular cell adhesion molecule-1 is expressed by cortical thymic epithelial cells and mediates thymocyte adhesion. Implications for the function of alpha4beta1 (VLA4) integrin in T-cell development", *Blood* **89**, 2461–2471.

Shortman, K., Egerton, M., Spangrude, G.J. and Scollay, R. (1990) "The generation and fate of thymocytes", *Semin. Immunol.* **2**, 3–12.

Smith, W.B., Gamble, J.R., Clark-Lewis, I. and Vadas, M.A. (1991) "Interleukin-8 induces neutrophil transendothelial migration", *Immunology* **72**, 65–72.

Springer, T.A. (1994) "Traffic signals for lymphocyte recirculation and leukocyte emigration: the multistep paradigm", *Cell* **76**, 301–314.

Suzuki, A., Andrew, D.P., Gonzalo, J.A., *et al.* (1996) "CD34-deficient mice have reduced eosinophil accumulation after allergen exposure and show a novel crossreactive 90-kD protein", *Blood* **87**, 3550–3562.

Vaday, G.G., Franitza, S., Schor, H., *et al.* (2001) "Combinatorial signals by inflammatory cytokines and chemokines mediate leukocyte interactions with extracellular matrix", *J. Leukoc. Biol.* **69**, 885–892.

Weber, C. and Springer, T.A. (1998) "Interaction of very late antigen-4 with VCAM-1 supports transendothelial chemotaxis of monocytes by facilitating lateral migration", *J. Immunol.* **61**, 6825–6834.

Worthylake, R.A. and Burridge, K. (2001) "Leukocyte transendothelial migration: orchestrating the underlying molecular machinery", *Curr. Opin. Cell Biol.* **13**, 569–577.

Xu, H., Gonzalo, J.A., St, P.Y., *et al.* (1994) "Leukocytosis and resistance to septic shock in intercellular adhesion molecule 1-deficient mice", *J. Exp. Med.* **180**, 95–109.

